# New Cretaceous (Aptian/Albian) boxer shrimp (Crustacea, Decapoda, Stenopodidea) from the Araripe Sedimentary Basin, NE, Brazil

**DOI:** 10.1371/journal.pone.0281334

**Published:** 2023-03-22

**Authors:** Damares Ribeiro Alencar, William Santana, Allysson Pontes Pinheiro, Daniel Lima, Antônio Álamo Feitosa Saraiva, Gustavo Ribeiro de Oliveira

**Affiliations:** 1 Department of Geology, Postgraduate Program in Geosciences (PPGEOC), Federal University of Pernambuco-UFPE, Recife, Pernambuco, Brazil; 2 Museum of Paleontology Plácido Cidade Nuvens, Santana do Cariri, Ceará, Brazil; 3 Department of Biological Sciences, Regional University of Cariri (URCA), Crato, Ceará, Brazil; 4 Department of Biology, Federal Rural University of Pernambuco (UFRPE), Recife, Pernambuco, Brazil; Sathyabama Institute of Science and Technology, INDIA

## Abstract

Stenopodidean shrimps are mostly cryptic in their habitats and are typically related with coral rubble or dead coral heads, rocks and crevices, and in association with other marine invertebrate such as sponges, crinoids and corals. Here, we describe a new stenopodidean shrimp, *Dubiostenopus parvus* n. gen. n. sp., from the Romualdo Formation (Aptian/Albian), Araripe Sedimentary Basin. The specimen studied here was collected in the municipality of Trindade, Pernambuco State, Brazil. The specimen is the imprint of a small shrimp approximately 10 mm in length, with a robust cephalothorax, a well-developed cheliped in the third pereiopod, and a second pleura not overlapping the first. This is the first stenopodidean shrimp described from the Romualdo Formation and the first described from South America. Comparisons with other Brazilian shrimp-like fossils are made, as well as comparisons with all other fossil stenopodideans.

## Introduction

Stenopodidea is a small group of shrimp-like marine decapods, with 92 extant species in a recent checklist [1]. Even being a small group, the taxonomy is intricate and the separation between families is troublesome [1,2]. As for the recent species, the fossil record of this group is scarce, with only four fossil species known worldwide. Nevertheless, the fossil records are very old dating back to the Devonian [3]. *Devonostenopus pennsylvaniensis* [3], from the Devonian of Pennsylvania, USA, *Jurastenopus frattigianii* [4], from the Upper Jurassic of the Solnhofen Lithographic Limestones of Germany, *Phoenice pasinii* [5], from the Cenomanian of Lebanon, and *Jilinocaris chinensis* [6], from the Santonian of China are the fossil species included in Stenopodidea. However, [4], suggested that *P*. *pasinii* should be excluded from Stenopodidea. In recent years, our group has studied several new species of decapods in the Araripe Sedimentary Basin, a well-known region for its quality and quantity of fossil findings [7,8]. Most of these findings are of shrimps, such as the caridean *Kellnerius jamacaruensis* [9]; the penaeoideans *Araripenaeus timidus* [10], and *Priorhyncha feitosai* [11]; and the luciferid *Sume marcosi* [12].

Within this framework, we describe a new stenopodidean species from the Romualdo Formation. The new species, *Dubiostenopus parvus* n. gen. n. sp., is illustrated and compared with other known fossil and recent species of stenopodideans. Although a familial assignment for the new species is impaired due to the condition of the fossil, we discuss the possible family assignment for the new species.

### Geological setting

The Araripe Basin is an important fossiliferous region, with high-quality, abundant records of several fossil groups. This basin is located between the states of Ceará, Pernambuco and Piauí, in the northeast region of Brazil. This is one of Brazil’s largest interior basins, covering an area of approximately 12,000 km^2^ [13]. Geologically, the Araripe Basin is formed of nine geological formations deposited in the pre-rift, rift, post-rift I and post-rift II periods, from the Jurassic to Cretaceous periods [14,15]. According to the stratigraphic proposal adopted here and previously established by [16], the Santana Formation was elevated to the category of the Santana Group, which includes the Barbalha, Crato, Ipubi and Romualdo formations.

The specimen described here was collected from the Romualdo Formation, which is formed of shales, sandstones, limestones, marls and carbonate concretions, with predomination of concretions interspersed between the shale levels [15]. The fossiliferous record of this formation is vast and diverse, including plants, vertebrates (e.g., fish, crocodilomorphs, dinosaurs, pterosaurs, and turtles) and invertebrates (echinoderms, mollusks, and pancrustaceans) [17] of unique preservation, attributing to this formation the designation of *Konservat Lagerstätte* [18]. This formation has been considered a transgressive marine environment, associated with strong marine incursions that occurred during the Cretaceous period [19].

## Materials and methods

**Fig 1:**
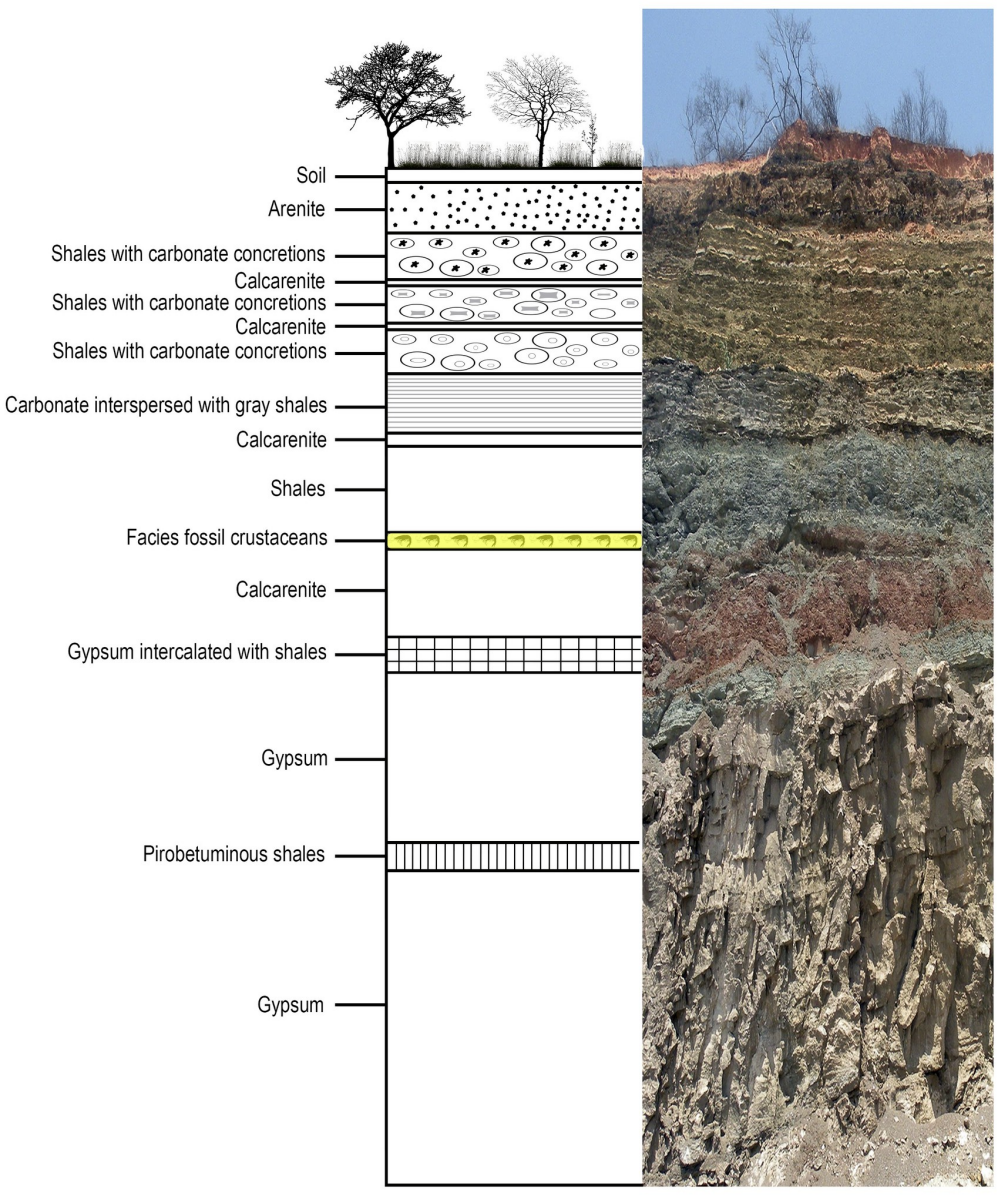
Stratigraphic profile and scheme of the Romualdo Formation. Stratigraphic profile and scheme of the Romualdo Formation (Cretaceous—Aptian/Albian) where the specimens were collected. Vertical exposure = 32 m.

**Fig 2:**
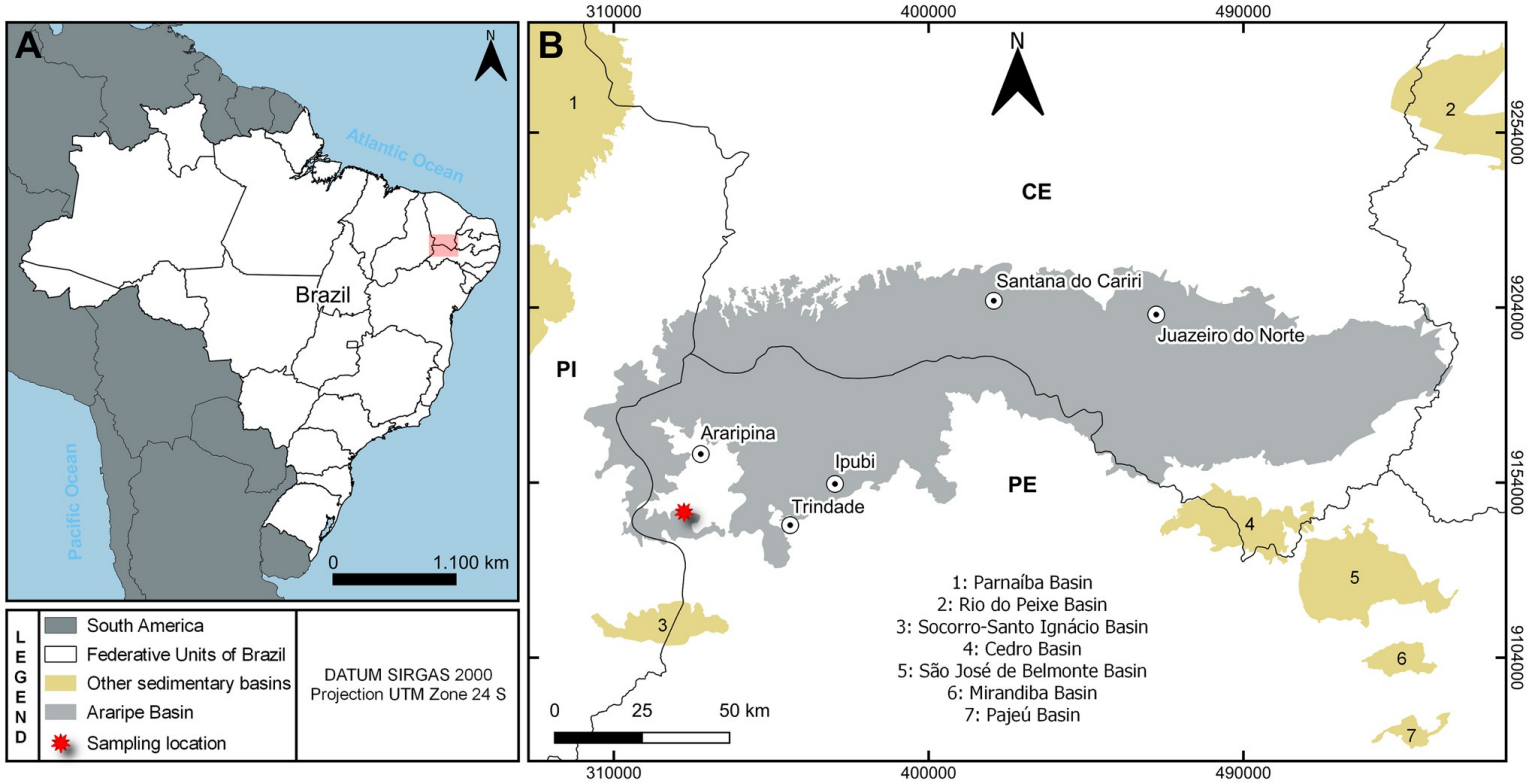
Geographic position of Trindade, Pernambuco, Brazil. Geographic position of the sampling site Trindade in the Araripe Basin, northeast Brazil. Colors indicate the different sequences of the Araripe Basin. Map figures (A) made with Natural Earth, and (B) was obtained from Brazilian Geological Service (CPRM, at https://geoportal.cprm.gov.br/geosgb/) powered by ESRI.

The material studied here was collected in the outcropping levels of dark shales from the Romualdo Formation (Fig 1) in a mining site (Mineradora Serra Suposta) of the municipality of Trindade, in the western region of the Pernambuco state, 07°43’37.4’’S, 040°32’26.8’’W (Fig 2).

We found this new species in the same site where we collected the type specimen of the planktic shrimp *Sume marcosi* [13]. It is represented by a single fossil specimen that was mechanically prepared and coated with Paraloid B-72. Descriptions, drawings and photographs were made using a stereomicroscope Nikon SMZ 745T equipped with camera lucida and a Leica EZ4 W, both with digital cameras attached. The software ISCapture 3.6.1 was used to take the measurements, all in millimeters (mm). In the descriptions, P1 is the first and P2 is the second pereiopod, and P3 to P5 are the third to fifth pereiopods, respectively. The specimen is deposited in the carcinological collection of the Museu de Paleontologia Plácido Cidades Nuvens (MPPCN) in Santana do Cariri, Ceará State, Brazil.

For morphological nomenclature we followed [20]. e.g., pereiopods instead of pereopods). The classification of stenopodideans mostly follows [21] and [22] although those works are based only on extant taxa. There is a confusion in the literature about the authority of the name Stenopodidea, being attributed to [23] or [24] in different works. For that matter we follow the work of Burkenroad [25] in which Spence Bate [24] is designated the author of this taxon.

All maps were created using QGIS software (version 3.16.16). Map figures from Fig 2A were made with Natural Earth. Free vector and raster map data @ naturalearthdata.com. Map figure from 2B were obtained from Brazilian Geological Service (CPRM, at https://geoportal.cprm.gov.br/geosgb/) powered by ESRI.

### Nomenclatural acts

The electronic edition of this article conforms to the requirements of the amended International Code of Zoological Nomenclature, and hence the new names contained herein are available under that Code from the electronic edition of this article. This published work and the nomenclatural acts it contains have been registered in ZooBank, the online registration system for the ICZN. The ZooBank LSIDs (Life Science Identifiers) can be resolved and the associated information viewed through any standard web browser by appending the LSID to the prefix “http://zoobank.org/”. The LSID for this publication is: urn:lsid:zoobank.org:pub:BC73C736-9D84-4C88-ABE9-D6EB140695D7. The electronic edition of this work was published in a journal with an ISSN, and has been archived and is available from the following digital repositories: LOCKSS (added: Sep 10 2012 6:55PM UTC) [http://www.lockss.org]; PubMed Central (added: Sep 10 2012 6:55PM UTC) [http://www.ncbi.nlm.nih.gov/pmc].

## Results

### Systematic paleontology

Decapoda Latreille, 1802

Pleocyemata Burkenroad, 1963

Stenopodidea Spence Bate, 1888

*Incertae sedis*

#### Remarks

The assignment to Stenopodidea is based in the following characters: (i) carapace more or less cylindrical; (ii) pleura of anterior pleomeres usually not expanded (second never overlapping first); (iii) and an enlarged third pereiopod. Also, the presence of teeth in the pleura ventral margins of the second to fifth pleomeres, not commonly seen in other groups, such as carideans, is another indicative of a stenopodidean position.

Characteristics used to differentiate between families, such as cutting edges of chelae, first pereiopod carpo-propodal setiferous organ, third maxillipeds, branchial formula, and telson morphology are characters not preserved in the present material; however, as suggested by [2] there is only one family in this infraorder, thus, the new species can be assigned to the family Stenopodidae.

### *Dubiostenopus* gen. nov.

LSID urn:lsid:zoobank.org:act:1BABA30F-1163-491F-9355-7E2F11C9645E

Type species. ***Dubiostenopus parvus* gen. nov. sp. nov**.

#### Diagnosis

Carapace thick; rostrum apparently short, with five dorsal short spines, diminishing in size anteriorly. Enlarged P3 chelated, strong. Ventral margin of second to fifth pleomeres with few, strong teeth.

#### Etymology

The genus name is composed of the Latin word “dubious,” meaning doubtful or uncertain, and “stenopus,” referring to the Stenopodidea infraorder in which it is placed. Gender masculine.

#### Description

Same as for the species.

#### Remarks

As several of the shrimp-like fossils of the Araripe Basin, the preservation quality of the material studied here make comparisons somehow difficult; however, *Dubiostenopus* n. gen. can be distinguished from *Jilinocaris* [6], *Phoenice* [5], and *Devonostenopus* [3] by the presence of strong teeth on the pleura ventral margin of the second to fifth pleomeres, sixth pleomere with smooth margins (teeth apparently not present in *Jilinocaris*, and *Phoenice*, the posterolateral margins of pleura 2–5 with one posteriorly oriented, acute, triangular spine in *Devonostenopus*). The separation of *Dubiostenopus* n. gen. from *Jurastenopus* [4] can be done by the number of dorsal spines in the rostrum (five in *Dubiostenopus* n. gen. and 16 dorsal and 3 ventral in *Jurastenopus*). Nevertheless, the preservation quality makes the number of dorsal spines in rostrum not a definitive character (i.e., the rostrum may be broken and the number of spines could be higher).

The characteristics observed in this new genus allow us to easily separate it from the previous known shrimp species from the Araripe Sedimentary Basin and other Brazilian fossil shrimps. The combination of an enlarged chelated P3, strong, and ventral margin of second to fifth pleomeres with strong teeth are only observed in *Dubiostenopus* n. gen.

### *Dubiostenopus parvus* n. gen. n. sp. (Fig 3)

**Fig 3:**
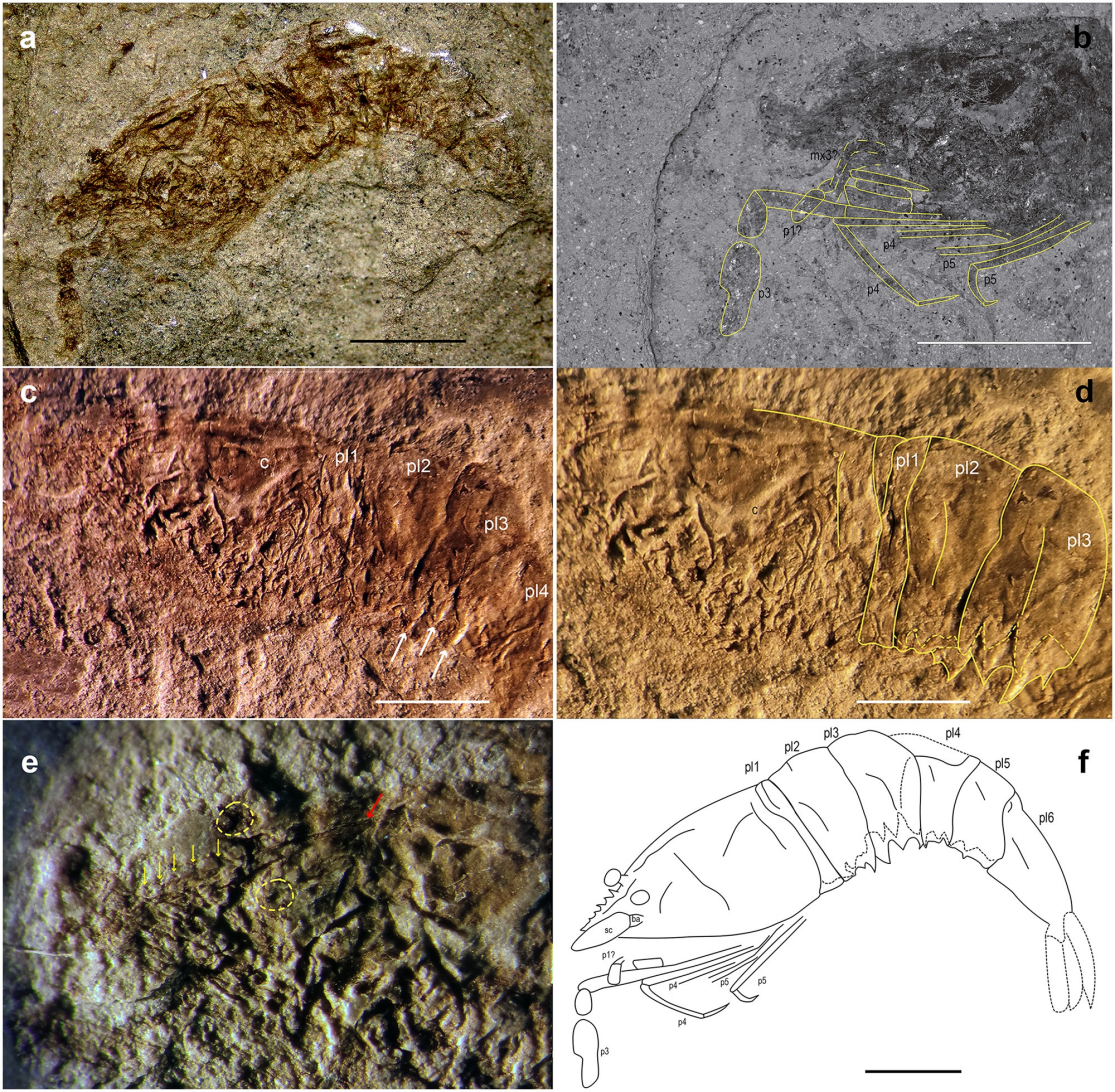
*Dubiostenopus parvus* n. gen. n. sp. *Dubiostenopus parvus* gen. nov. sp. nov. (MPSC 2542) from the Romualdo Formation, Cretaceous (Aptian/Albian), Araripe Basin: (a) lateral view of the holotype; (b) SEM image of the holotype, lateral view, detail of pereiopods; (c) detail of pleomeres 1–4; white arrows show the third pleura strong ventral spines; (d) detail of pleomere 1–3; dotted lines represent the right pleural margins; (e) detail of anterior region; dotted lines represent the eyes position; yellow arrows indicate the dorsal rostral spines; red arrow indicates the rostrum posterior margin; (f) line drawing of the holotype; dotted lines represent pl4 dorsal region not preserved and the right pleural margins. Scale bars: 2 mm. Specimen dry, uncoated. Photos: (a, b) A.P. Pinheiro; (c, e, f) D. Lima. Line drawing by D. Lima.

LSID urn:lsid:zoobank.org:act:D44C3015-DF9A-4208-9DD3-FC1776DE61D0

#### Holotype

The single specimen MPSC 2542, without counterpart.

#### Type Locality

Trindade municipality, western region of the Pernambuco State, Brazil.

#### Stratigraphic Unit and Age

Romualdo Formation, Santana Group, Araripe Sedimentary Basin; Aptian/Albian.

#### Diagnosis

Same as for the genus.

#### Etymology

‘parvus’ from the Latin means little, small, alluding to the reduced size of this species.

#### Occurrence

Dark shales of the Romualdo Formation, Araripe Sedimentary Basin.

#### Description

Fossil imprint preserved in lateral view, slightly twisted laterally. Total length from anterior margin of carapace to the posterior margin of sixth pleonal segment 10 mm. Carapace robust, slightly rounded, wide posteriorly, tapering anteriorly, with ventrolateral angle rather rounded; cervical groove well-marked, long, reaching almost carapace lateral margin, branchiostegal groove discernible, apparently short, ending before the cervical groove, intestinal groove parallel to the posterior margin extending to almost middle of carapace; no spines or tubercles observed. Eyes clearly discernible as rounded darker spots, with short eyestalks. Rostrum slightly shorter than scaphocerite, apparently straight, dorsal margin with five short spines, tapering distally, no signs of lateral spines. Basicerite and scaphocerite of the left antennae preserved, other parts of antennae and antennulae not preserved. Third maxilliped fragments indistinctly preserved among other pereiopod parts. First and second pereiopods preserved as indistinct fragments near frontal region. P3 very long, chelate, with movable finger (dactylus) not preserved, propodus (fixed finger) inflated, longer than carpus, merus slender, division between merus, ischium, basis and coxa not discernible. Fourth pereiopod apparently complete, shorter than P3, longer than P5, slender, segmentation between propodus, carpus, merus and ischium not discernible, dactylus distinct; fifth pereiopod apparently complete, shorter than others, segmentation between propodus, carpus, merus and ischium not discernible, dactylus discernible. Pleomeres without ornamentation dorsally; pleomere 1 shorter, pleomere 6 longest; second pleomere slightly overlapping only third; first pleomere with a small groove dorsolaterally, ventral margin rounded; second pleomere with short dorsal groove and long lateral groove, ventral margin with 2 distinct teeth; third pleomere enlarged, with long lateral groove, ventral margin with 3 strong teeth; fourth pleomere dorsal margin damaged, with short lateral groove, ventral margin with 1 small anterior and 1 small posterior teeth; fifth pleomere with an oblique, rather short lateral groove, ventral margin with 1 small median tooth; sixth pleomere with an oblique, long anterolateral groove, ventral margin smooth. Pleopods, uropods and telson not preserved.

## Discussion

This is the first fossil representative of Stenopodidea found in the Araripe Basin, and in South America. The greatest number and diversity of modern stenopodidean species and genera is recorded in the Indo-West Pacific region, with a secondary radiation into the tropical western Atlantic, probably associated with the ancient Tethys Sea [22]. The quality of the preservation of the single specimen of *Dubiostenopus parvus* n. gen. n. sp. is not as good as observed in other species found in the Araripe Sedimentary Basin, such as specimens of *Beurlenia araripensis* [26,27]. Nevertheless, *Dubiostenopus parvus* n. gen. n. sp. is different from other shrimp-like fossils known from Brazil.

Characteristics, such as the short rostrum with five dorsal spines, tapering distally and a strong chelated third pereiopod clearly separate *Dubiostenopus parvus* n. gen. n. sp. from the caridean *Kellnerius jamacaruensis*, the other shrimp found in the Romualdo Formation that presents a long rostrum with strong spines dorsally, without spines distally, and delicate pereiopods. *Dubiostenopus parvus* n. gen. n. sp. is also distinct from *Bahiacaris roxoi* [28], another caridean from the Cretaceous of the Marizal Formation (Bahia State, Brazil) that presents a long rostrum with a serrulate dorsal margin and pereiopods with similar sizes (rostrum with five spines and third pereiopod much longer, with fourth smaller and fifth the smallest in *D*. *parvus* n. gen. n. sp.).

Different from *D*. *parvus* n. gen. n. sp., which is found in the Romualdo Formation—a lagoon environment thought to have had high salinity [29]—*Beurlenia araripensis* is a freshwater shrimp from the Crato Formation and a well-known species with several described specimens. Although *B*. *araripensis* presents strong chelipeds, those are the P1 and P2, P2 being the strongest (third pereiopod with strongest cheliped in *D*. *parvus* n. gen. n. sp.). Also, *B*. *araripensis* is a much larger species, with different degrees of ornamentation of the rostrum [27] and well-developed eyestalks, differing considerably from *Dubiostenopus parvus* n. gen. n. sp. Other fossil shrimps from Brazil are from different epochs than *Dubiostenopus parvus* n. gen. n. sp. [29] and do not present such characteristics as a strong and distinct cheliped in the third pereiopod.

The presence of the a stenopodidean boxer shrimp from Romualdo Formation is a strong indicative of a shallow marine warm water paleoenvironment, which corroborates with the hypothesis on the formation of a substantial epicontinental sea at the Araripe Basin during the Aptian age [30,31], once all fossil and extant species of these relatively small infraorder live in marine environments. The shallow water species generally can be found in crevices and overhangs of coral reefs or in association with other invertebrates, such as hexactinellid sponges [22]. The small size and the apparently lacking of spines in the carapace, pleon and appendages in *D*. *parvus* n. gen. n. sp. may be an indicative of an adaptation to a commensal or symbiotic life with hexactinellid sponges as seen in many spongicolids [4] and references therein.

### Fossil in the Araripe Unesco Global Geopark

The increasing diversity of the fossil shrimps, and other decapod groups from the Araripe Basin is a result of the efforts of several institutions in the last years, such as the Universidade Regional do Cariri and the Araripe Unesco Global Geopark, which have implemented programs of geoconservation and geoeducation for the local community.

The result presented in this work is a direct reflect of those actions, which led people from the community to send the material to be studied, instead of selling it for fossil dealers (9–13). The deposition of the material also promotes the Museu de Paleontologia Plácido Cidade Nuvens and inspire the inhabitants of the Araripe territory to maintain their heritage, which shapes the identity of the people and their feeling of belonging and culture.
